# Supplementary material for: Disrupting HIV‐1 capsid formation causes cGAS sensing of viral DNA
Source: EMBO J. 2020 Aug 27;39(20):e103958. doi: 10.15252/embj.2019103958 (PMC7560218; doi:10.15252/embj.2019103958)
Supplement: Supplementary file 1 — Appendix [file EMBJ-39-e103958-s001.pdf]

Appendix to:

Disrupting HIV-1 capsid formation causes cGAS sensing of viral DNA

Rebecca P. Sumner*, Lauren Harrison, Emma Touizer, Thomas P. Peacock, Matthew Spencer, Lorena Zuliani-Alvarez & Greg J. Towers

* Corresponding author. Correspondence: r.sumner@ucl.ac.uk

This appendix includes:

Appendix figure S1: Generation of THP-1 cell lines depleted for SAMHD1
Appendix figure S2: IFN blockade does not rescue suppression of spreading infection by LPV in MDM
Appendix figure S3: Gag-defective HIV-1 particles are less able to saturate restriction factor TRIM5
Appendix figure S4: The HIV-1 capsid protects viral DNA from sensing by cGAS

Appendix figure S1: Generation of THP-1 cell lines depleted for SAMHD1

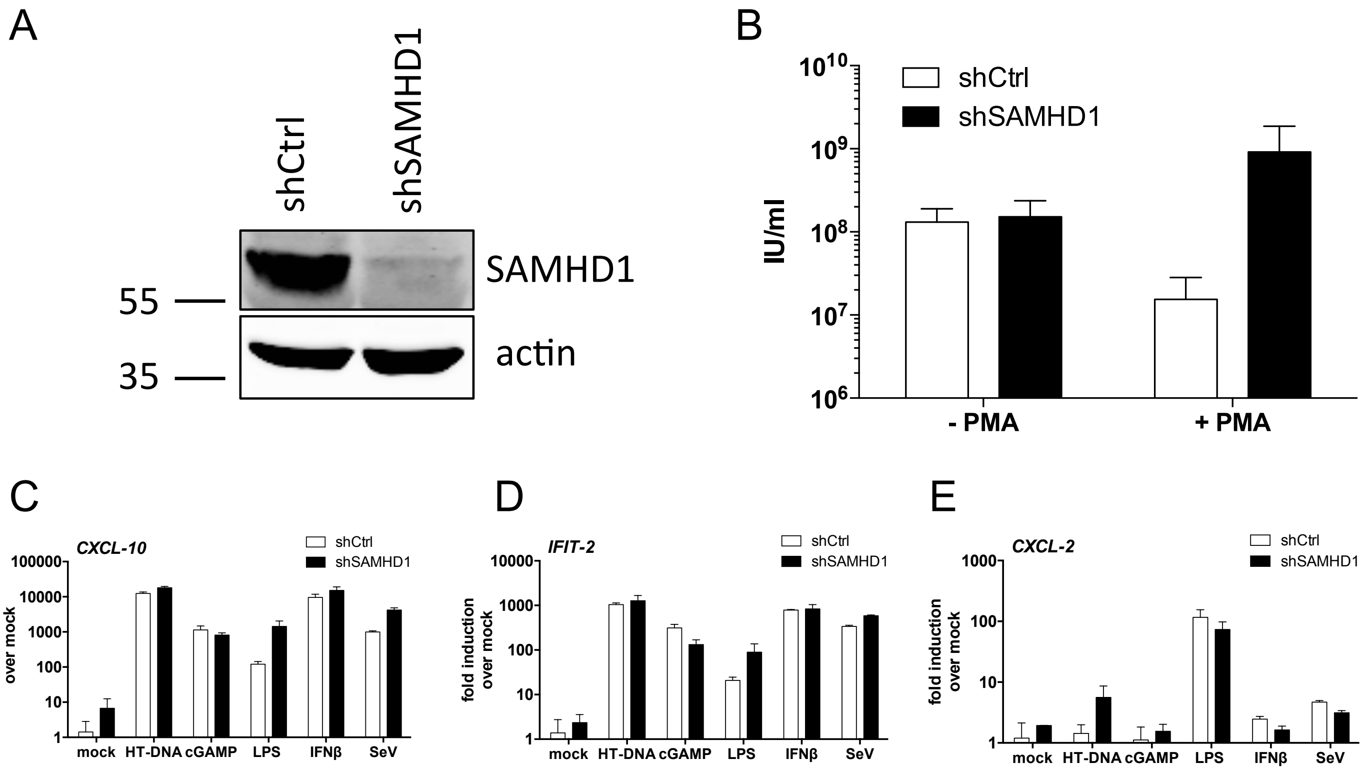

Appendix Figure S1. Generation of THP-1 cell lines depleted for SAMHD1. (A) Immunoblot of THP-1 cell line with stable depletion of SAMHD1 (shSAMHD1) or a corresponding control shRNA (shCtrl) (see Methods). Blots were probed with anti-SAMHD1 and anti-actin antibodies. Molecular mass markers (in kDa) are indicated on the left. (B) Titration of HIV-1 GFP on THP-1 shCtrl and THP-1 shSAMHD1 cells that had been treated or not with PMA (50 ng/ml) for 48 h. Infectious units per ml (IU/ml) were calculated by enumeration of GFP-positive cells by flow cytometry. Data are mean ± SD from three separate titrations performed in singlet. (C-E) ISG qPCR from PMA-treated THP-1 shCtrl and THP-1 shSAMHD1 cells that had been stimulated for 8 h with HT-DNA (20 ng/ml, transfected), cGAMP (1 µg/ml), LPS (1 µg/ml), IFNβ (10 ng/ml) or Sendai virus (SeV) (0.2 HA U/ml). Data are mean ± SD, n=2, representative of 2 repeats.

Appendix figure S2: IFN blockade does not rescue suppression of spreading infection by LPV in MDM

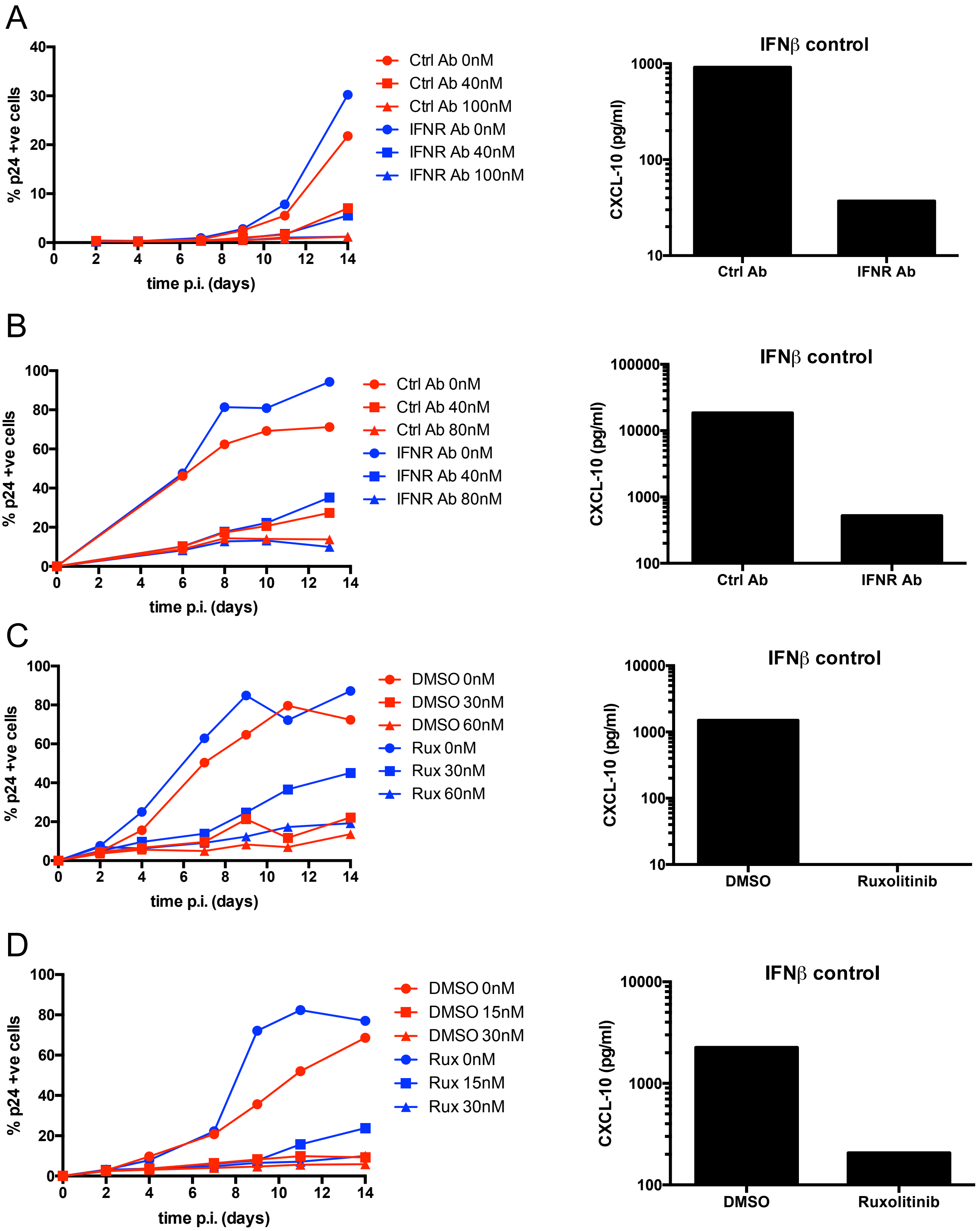

Appendix Figure S2. IFN blockade does not rescue suppression of spreading infection by LPV in MDM. Spreading infection of R9 BaL (0.02U RT per well of a 24wp) on primary MDM in the presence of varying concentrations of LPV as indicated (0-100 nM) and in the presence or absence of 1 µg/ml IFN receptor blocking Ab (IFNR Ab, A&B) or 2 µM ruxolitinib (C&D). LPV, blocking Abs and ruxolitinib were replaced every 3-4 days. Percentage infected cells (left) was calculated by staining cells with an anti-p24 antibody and analysing by flow cytometry. To confirm effectiveness of the IFNR Ab and ruxolitinib cells were stimulated in parallel with 1 ng/ml IFNβ and CXCL-10 in the supernatant was measured 48h later by ELISA (right). Data are from a single well at each time point.

Appendix figure S3: Gag-defective HIV-1 particles are less able to saturate restriction factor TRIM5

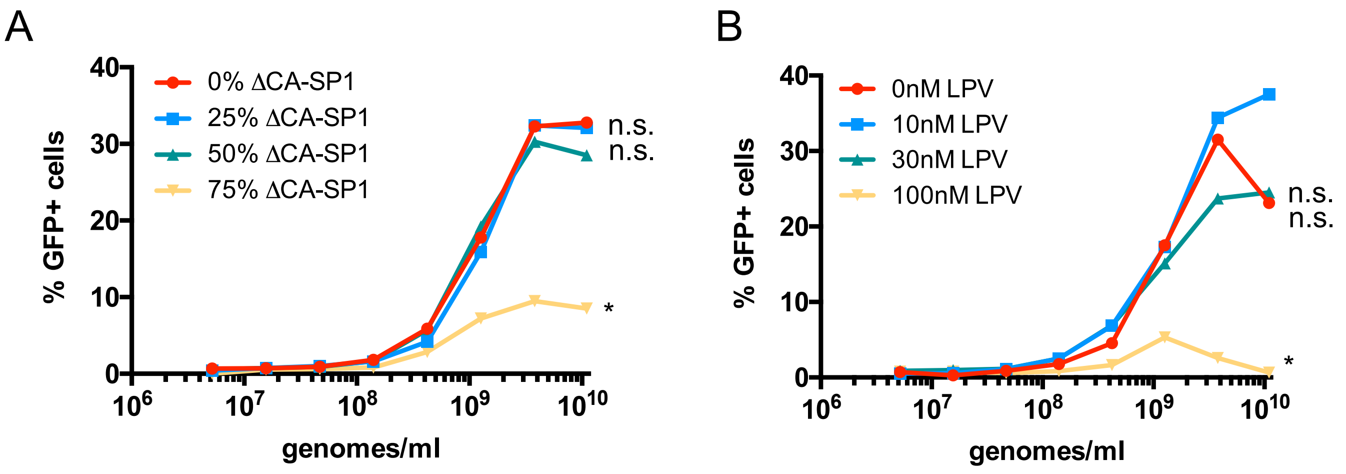

Appendix figure S3. Gag-defective HIV-1 particles are less able to saturate restriction factor TRIM5. Abrogation-of-restriction assay in FRhK4 cells expressing restrictive rhesus TRIM5. (A) Repeat assay of data presented in Fig. 5A. FRhK4 cells were co-transduced with a fixed dose of HIV-1 GFP (5×10^7 genomes/ml) and increasing doses of HIV-LUC ΔCA-SP1 mutants as indicated (5.2×10^6 - 1.1×10^{10} genomes/ml). (B) Repeat assay of data presented in Fig. 5B. FRhK4 cells were co-transduced with a fixed dose of HIV-1 GFP (5×10^7 genomes/ml) and increasing doses of LPV-treated HIV-LUC viruses as indicated (5.2×10^6 - 1.1×10^{10} genomes/ml). Rescue of GFP infectivity was assessed by flow cytometry. Data are singlet % GFP values. Statistical analyses were performed using 2-way ANOVA with multiple comparisons. * $P<0.05$.

Appendix figure S4: The HIV-1 capsid protects viral DNA from sensing by cGAS

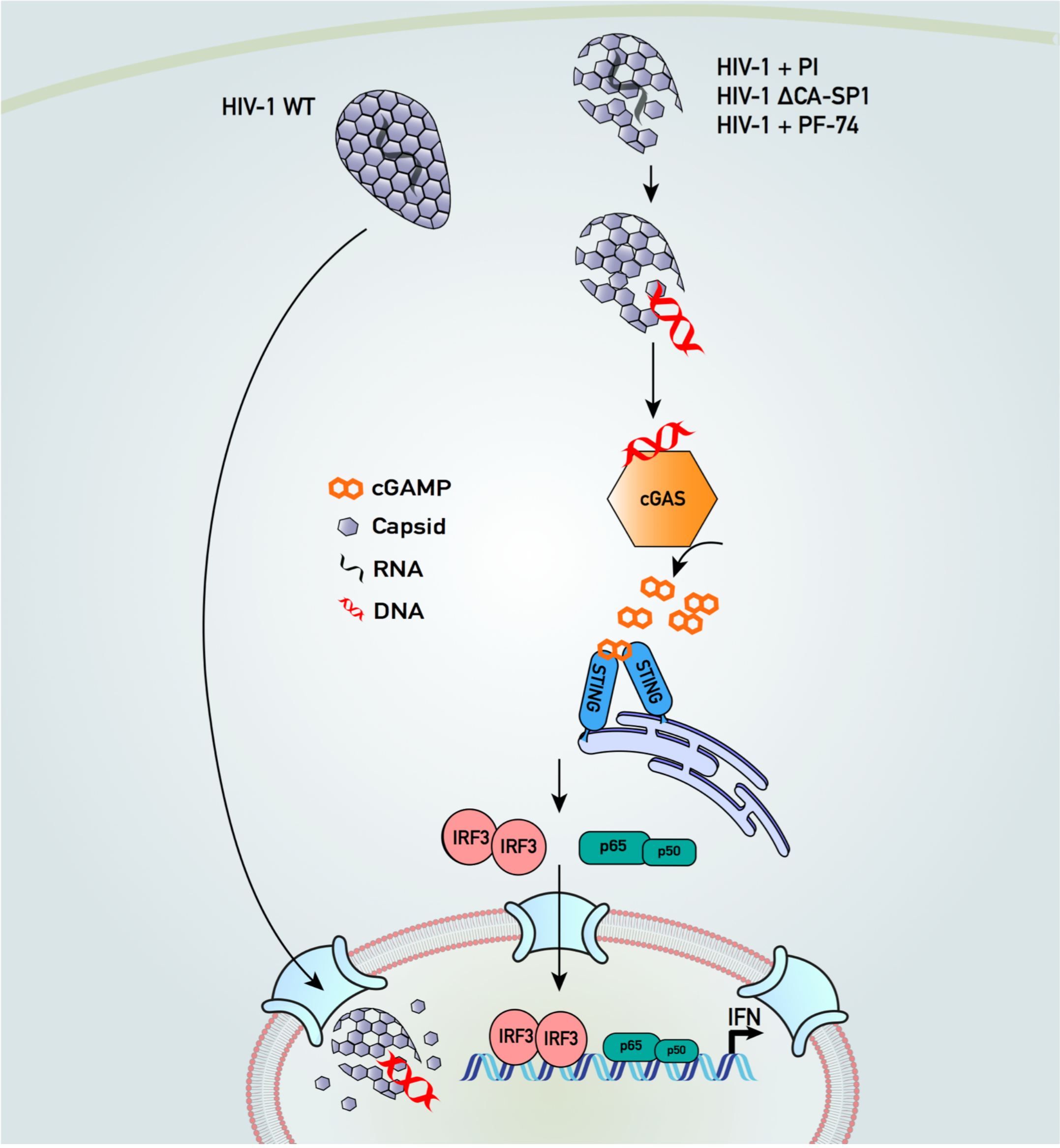

Appendix Figure S4. Disrupting HIV-1 capsid formation causes cGAS sensing of viral DNA.
After entry wild-type (WT) HIV-1 stays intact as it traverses the cytoplasm allowing it to synthesise its DNA without activating a type I IFN response. Conversely treatment of HIV-1 with protease inhibitors (PI), capsid destabilising small molecule PF-74 or mutation of the protease cleavage site between capsid and spacer peptide 1 (HIV-1 ΔCA-SP1) leads to defective particles that fail to protect viral DNA from innate sensor cGAS. Binding of cGAS to viral DNA leads to the production of cGAMP that binds STING and stimulates IFN production through activation of the transcription factors IRF3 and p50/p65 (NF-κB).
