# Supplementary material for: Disrupting HIV‐1 capsid formation causes cGAS sensing of viral DNA
Source: EMBO J. 2020 Aug 27;39(20):e103958. doi: 10.15252/embj.2019103958 (PMC7560218; doi:10.15252/embj.2019103958)
Supplement: Supplementary file 2 — Expanded View Figures PDF [file EMBJ-39-e103958-s002.pdf]

# Expanded View Figures

**Figure EV1. PI treatment induces HIV-1 to trigger an ISG response in macrophages.**

A Schematic of intermediate Gag cleavage products. MA: matrix, CA: capsid, SP1: spacer peptide 1, NC: nucleocapsid, SP2: spacer peptide 2.
B Infection data from Fig 1D–G. PMA-treated THP-1 shSAMHD1 cells transduced for 48 h with LPV-treated HIV-1 GFP viruses (0.1 U RT/ml or 0.5 U RT/ml).
C Immunoblot of HIV-1 GFP virus particles ($2 \times 10^{11}$ genomes) produced with darunavir (DRV, 0–50 nM) detecting p24.
D Titration of DRV-treated HIV-1 GFP viruses on U87 cells. Mean ± SD, $n = 3$.
E Infection data from (F, G).
F IRF reporter activity from PMA-treated THP-1 Dual shSAMHD1 cells transduced for 24 h with DRV-treated HIV-1 GFP ($1 \times 10^{10}$ genomes/ml).
G CXCL-10 protein in supernatant from (F) (ELISA).
H ISG qRT-PCR from PMA-treated THP-1 shSAMHD1 cells transduced for 24 h with 0.2 U RT/ml 30 nM LPV-treated HIV-1 GFP in the presence of DMSO vehicle or 2 μM ruxolitinib. A control was stimulated with 1 ng/ml IFNβ.
I, J Titration of LPV-treated HIV-1 R9 BaL viruses on primary MDM. Collated data (mean ± SD, $n = 3$) represented as percentage titre normalised to R9 BaL produced in the absence of LPV (0 nM) are in (I) and data from individual donors are in (J).
K CXCL-10 protein in supernatant of primary MDM 48 h post-transduction with WT HIV-1 GFP or DRV-treated (12.5 nM) HIV-1 GFP ($6 \times 10^7$ genomes/ml or $3 \times 10^8$ genomes/ml) in the presence of DMSO vehicle or 2 μM ruxolitinib.
L Infection data from (K).

Data information: Data are mean ± SD, $n = 3$, representative of 2 repeats (E–H, K, L) or 3 repeats (B). Statistical analyses were performed using the Student's *t*-test, with Welch's correction where appropriate and comparing to the 0 nM DRV condition. *$P < 0.05$, **$P < 0.01$.
Source data are available online for this figure.

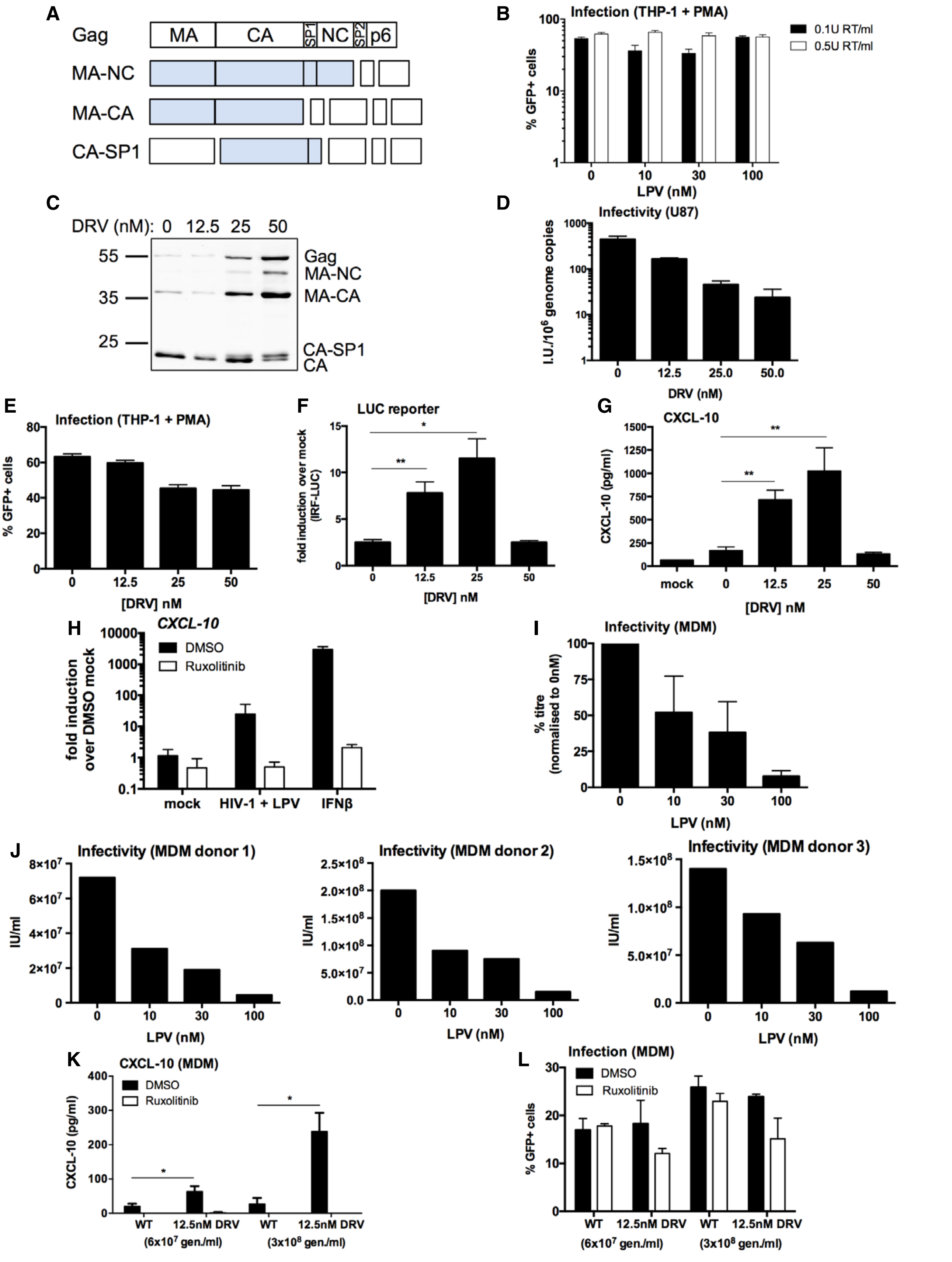
A
Gag
MA
CA
SP1
NC
SP2
p6
MA-NC
MA-CA
CA-SP1
B
Infection (THP-1 + PMA)
0.1U RT/ml
0.5U RT/ml
% GFP+ cells
LPV (nM)
C
DRV (nM): 0 12.5 25 50
55
35
25
Gag
MA-NC
MA-CA
CA-SP1
CA
D
Infectivity (U87)
I.U./10^6 genome copies
DRV (nM)
E
Infection (THP-1 + PMA)
% GFP+ cells
[DRV] nM
F
LUC reporter
fold induction over mock (IRF-LUC)
[DRV] nM
G
CXCL-10
CXCL-10 (pg/ml)
mock
[DRV] nM
H
CXCL-10
DMSO
Ruxolitinib
fold induction over DMSO mock
mock
HIV-1 + LPV
IFNβ
I
Infectivity (MDM)
% titre (normalised to 0nM)
LPV (nM)
J
Infectivity (MDM donor 1)
Infectivity (MDM donor 2)
Infectivity (MDM donor 3)
IU/ml
LPV (nM)
K
CXCL-10 (MDM)
DMSO
Ruxolitinib
CXCL-10 (pg/ml)
WT
12.5nM DRV
(6x10^7 gen./ml)
(3x10^8 gen./ml)
L
Infection (MDM)
DMSO
Ruxolitinib
% GFP+ cells

**Figure EV1.**

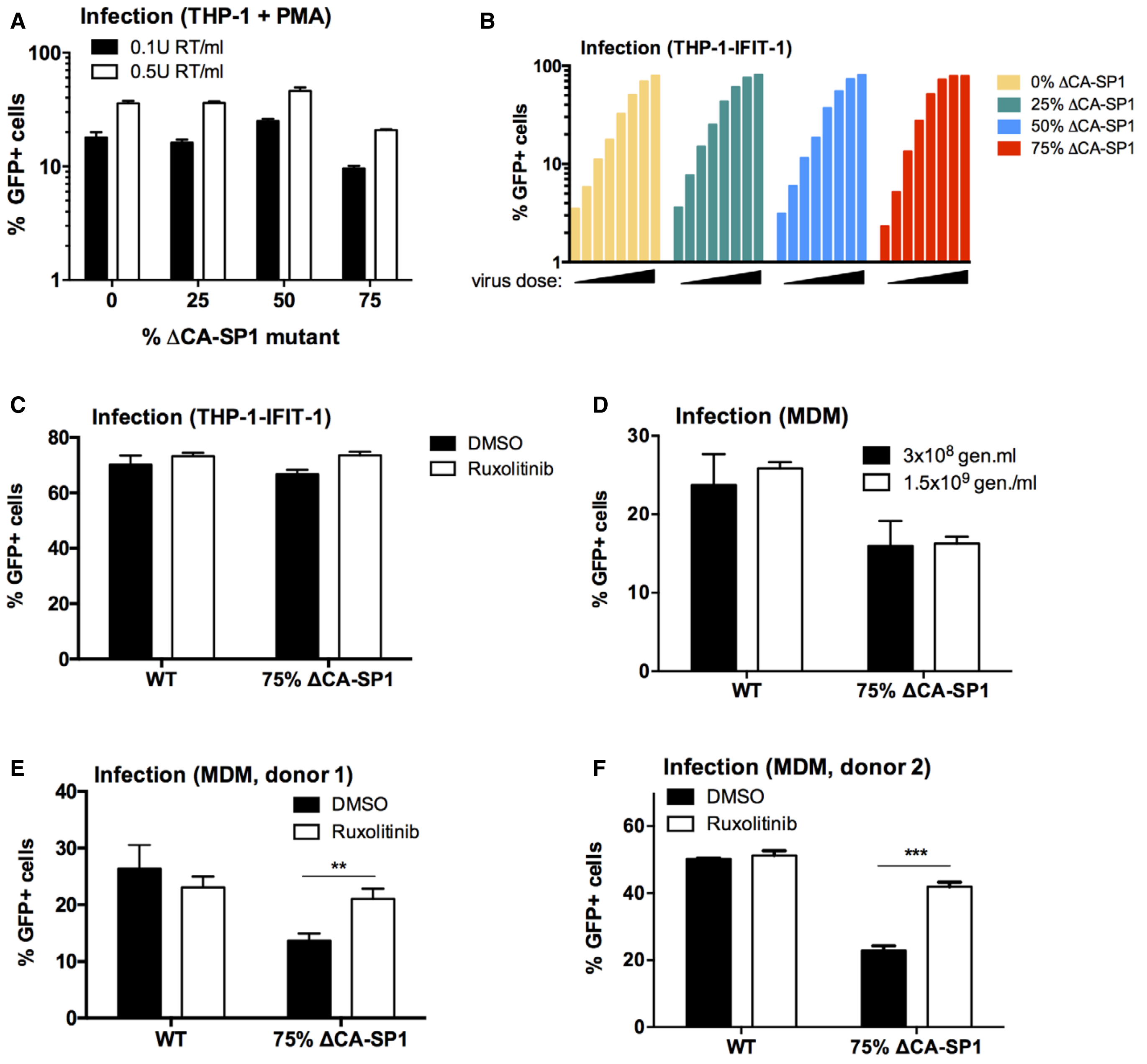


**Figure EV2. HIV-1 with Gag protease cleavage mutation induces ISGs in macrophages.**

A Infection levels of cells from Fig 2C–E. PMA-treated THP-1 shSAMHD1 cells transduced for 48 h with HIV-1 GFP ΔCA-SP1 viruses (0.1 U RT/ml or 0.5 U RT/ml).

B Infection data from Fig 2G. THP-1-IFIT-1 cells transduced for 48 h with HIV-1 GFP ΔCA-SP1 viruses (0.016–0.2 U RT/ml).

C Infection data from Fig 2H. THP-1-IFIT-1 cells transduced for 48 h with HIV-1 GFP containing either 0% (WT) or 75% ΔCA-SP1 mutant in the presence of DMSO vehicle or 2 μM ruxolitinib.

D Infection data from Fig 2I. Primary MDM transduced for 48 h with WT HIV-1 GFP or 75% ΔCA-SP1 mutant ($3 \times 10^8$ genomes/ml or $1.5 \times 10^9$ genomes/ml).

E Infection data from Fig 2J and K. Primary MDM transduced for 48 h with WT HIV-1 GFP or 75% ΔCA-SP1 mutant ($1.5 \times 10^9$ genomes/ml) in the presence of DMSO vehicle or 2 μM ruxolitinib.

F Repeat of (E) in a second donor.

Data information: Data are individual measurements (B) or mean ± SD, $n = 3$ (A, C–F), representative of 2 repeats. Statistical analyses were performed using Student's *t*-test, with Welch's correction where appropriate and comparing to the DMSO control. $^{**}P < 0.01$, $^{***}P < 0.001$.

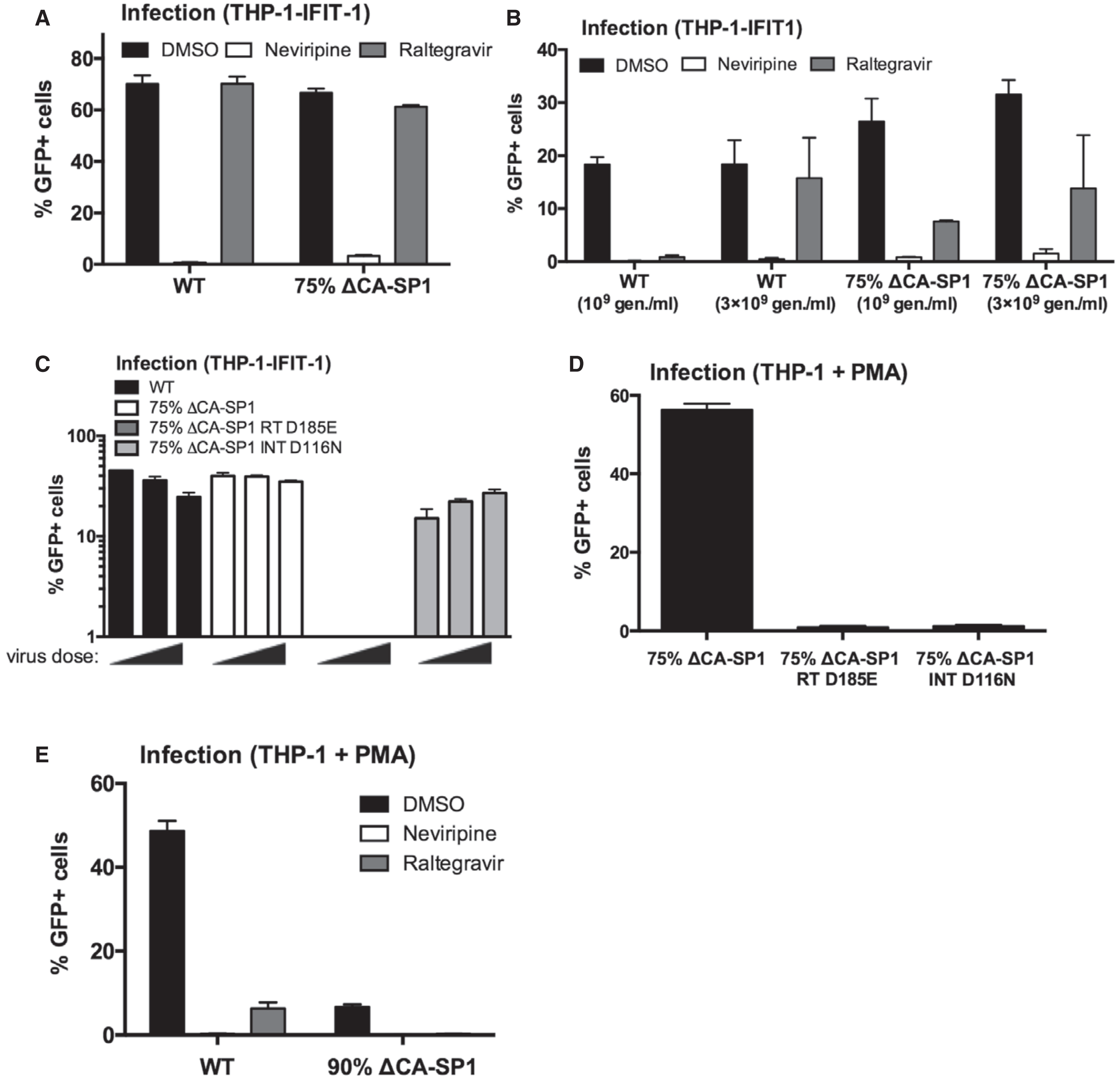


**Figure EV3. Innate immune activation is RT-dependent.**

A Infection data from Fig 3A and B. THP-1-IFIT-1 cells transduced for 48 h with HIV-1 GFP containing 0% (WT) or 75% ΔCA-SP1 mutant (1 U RT/ml) in the presence of DMSO vehicle, 5 μM nevirapine or 10 μM raltegravir.

B Infection data for Fig 3C and D. THP-1-IFIT-1 cells transduced for 48 h with 0% (WT) or 75% ΔCA-SP1 mutant ($10^9$ and $3 \times 10^9$ genomes/ml) in the presence of DMSO vehicle, 5 μM nevirapine or 10 μM raltegravir.

C Infection data for Fig 3E and F. THP-1-IFIT-1 cells transduced for 48 h with HIV-1 GFP containing 0% ΔCA-SP1 (WT), 75% ΔCA-SP1, 75% ΔCA-SP1 carrying a mutation in reverse transcriptase (75% ΔCA-SP1 RT D185E) or 75% ΔCA-SP1 carrying a mutation in integrase (75% ΔCA-SP1 INT D116N) ($3.75 \times 10^9$, $7.5 \times 10^9$ and $1.5 \times 10^{10}$ genomes/ml).

D Infection data for PMA-treated THP-1 Dual shSAMHD1 cells transduced for 48 h with 75% ΔCA-SP1, 75% ΔCA-SP1 RT D185E or 75% ΔCA-SP1 INT D116N ($3 \times 10^9$ genomes/ml).

E Infection data for PMA-treated THP-1 Dual shSAMHD1 control cells transduced for 48 h with WT HIV-1 GFP or 90% ΔCA-SP1 mutant ($1 \times 10^{10}$ genomes/ml) in the presence of DMSO vehicle, 5 μM nevirapine or 10 μM raltegravir.

Data information: Data are mean ± SD, $n = 3$, representative of 2 repeats.

 © 2020 The Authors

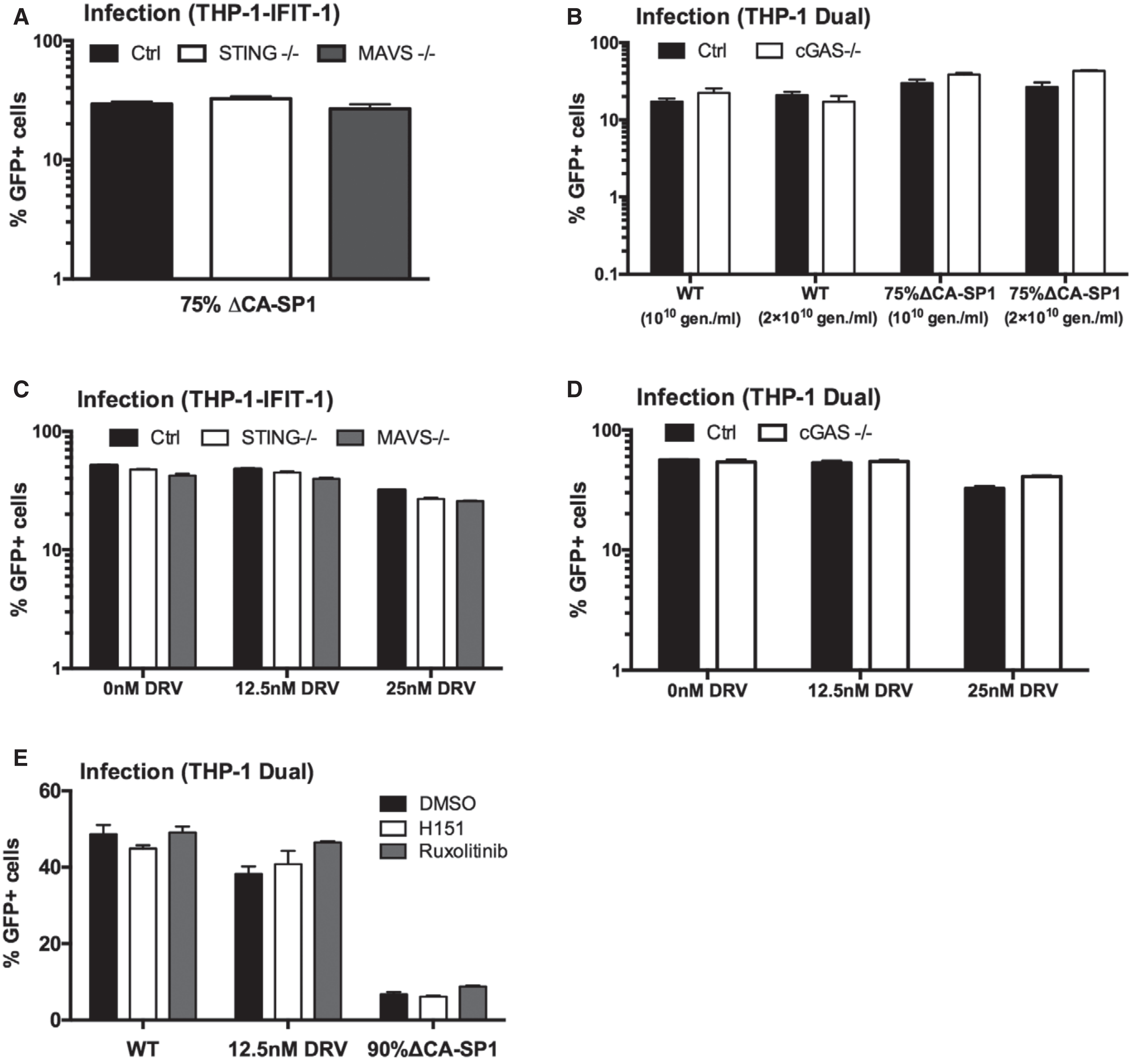


**Figure EV4. Innate immune activation is DNA sensing-dependent.**

A Infection data from Fig 4A. PMA-treated THP-1-IFIT-1 shSAMHD1 cells lacking STING or MAVS, or a gRNA control (Ctrl) cell line transduced for 48 h with HIV-1 GFP 75% ΔCA-SP1 (0.4 U RT/ml).

B Infection data from Fig 4C and D. PMA-treated THP-1 Dual shSAMHD1 cells lacking cGAS or a matching control (Ctrl) cell line transduced for 48 h with HIV-1 GFP containing either 0% (WT) or 75% ΔCA-SP1 ($1 \times 10^{10}$ and $2 \times 10^{10}$ genomes/ml).

C Infection data from Fig 4E and G. PMA-treated THP-1-IFIT-1 shSAMHD1 cells lacking STING, MAVS or matching gRNA control (Ctrl) cell lines transduced for 48 h with DRV-treated HIV-1 GFP ($1 \times 10^{10}$ genomes/ml).

D Infection data from Fig 4F and H. PMA-treated THP-1 Dual shSAMHD1 cells lacking cGAS or matching control (Ctrl) cell lines transduced for 48 h with DRV-treated HIV-1 GFP ($1 \times 10^{10}$ genomes/ml).

E Infection data from Fig 4I. PMA-treated THP-1 Dual shSAMHD1 control cells transduced for 48 h with WT, DRV-treated (DRV, 12.5 nM) or HIV-1 GFP 90% ΔCA-SP1 ($1 \times 10^{10}$ genomes/ml) in the presence of DMSO vehicle, 2 μM ruxolitinib or 0.5 μg/ml H151.

Data information: Data are mean ± SD, $n = 3$, representative of 2 (C–E) or 3 (A, B) repeats.

© 2020 The Authors

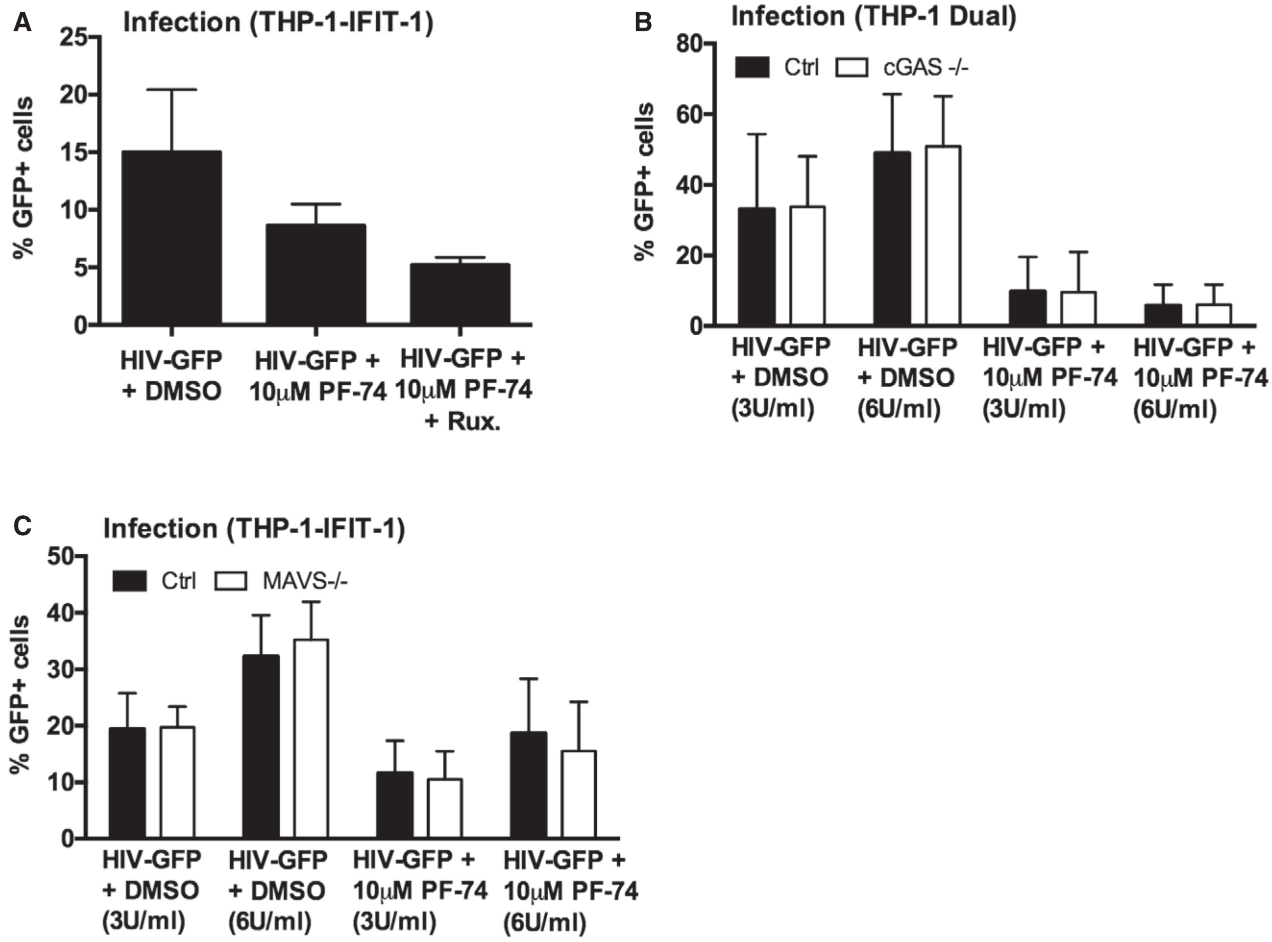


**Figure EV5. PF-74 treatment induces HIV-1 to trigger a DNA sensing-dependent ISG response.**

A Infection data from Fig 6F. THP-1-IFIT-1 cells transduced for 48 h with HIV-1 GFP (3 U/ml RT) in the presence of DMSO control or PF-74 (10 μM) and ruxolitinib (Rux, 2 μM) as indicated.

B Infection data from Fig 6G. THP-1 Dual shSAMHD1 cells lacking cGAS or a matching control (Ctrl) cell line transduced for 48 h with HIV-1 GFP (3 and 6 U/ml) in the presence of DMSO vehicle or PF-74 (10 μM).

C Infection data from Fig 6H. THP-1-IFIT-1 cells lacking MAVS or a matching gRNA control (Ctrl) cell line transduced for 48 h with HIV-1 GFP (3 and 6 U/ml) in the presence of DMSO vehicle or PF-74 (10 μM).

Data information: Data are mean ± SD, $n$ = 2 (B), 3 (A) or 4 (C), representative of 3 repeats.


© 2020 The Authors
