# Supplementary figures and images for: Disrupting HIV‐1 capsid formation causes cGAS sensing of viral DNA
Source: EMBO J. 2020 Aug 27;39(20):e103958. doi: 10.15252/embj.2019103958 (PMC7560218; doi:10.15252/embj.2019103958)

**Fig 1A**

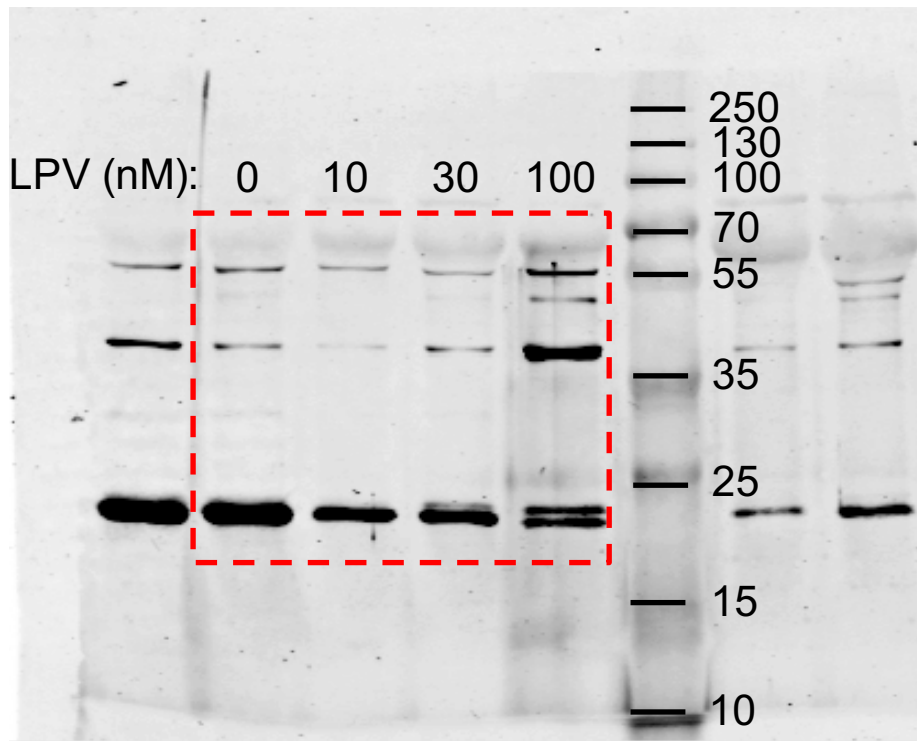

**Fig 1J**

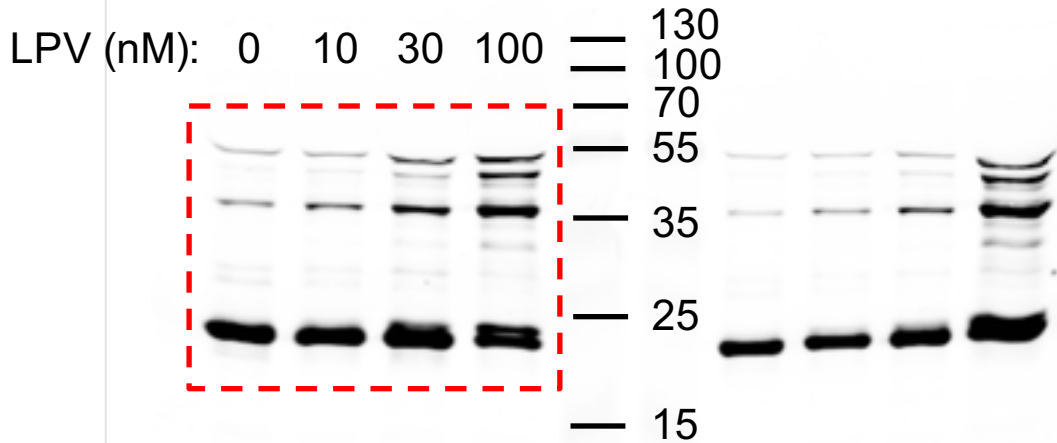

Supplement: Supplementary file 3 — Source Data for Figure 1 [file EMBJ-39-e103958-s003.pdf]

**Fig 2A**

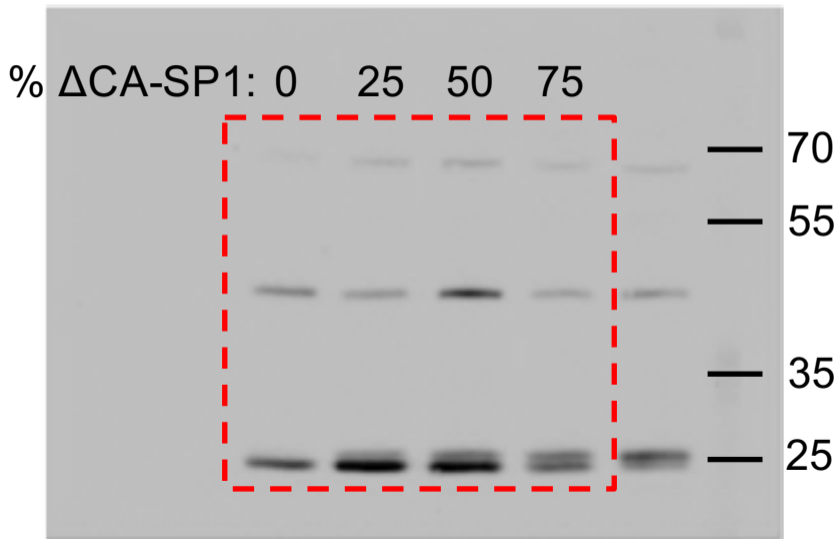

Supplement: Supplementary file 4 — Source Data for Figure 2 [file EMBJ-39-e103958-s004.pdf]

**Fig EV1C**

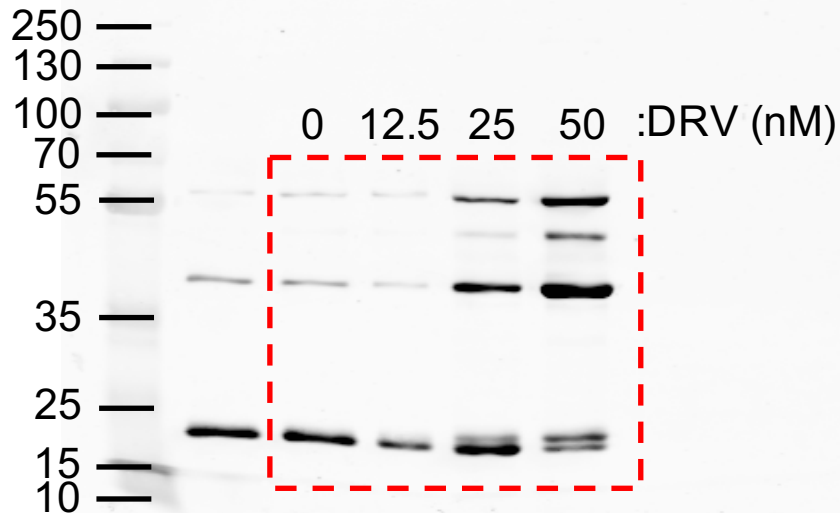

Supplement: Supplementary file 5 — Source Data for Expanded View [file EMBJ-39-e103958-s005.zip › embj2019103958-sup-0005-SDataFigEV1C.pdf]
